# Impact of Musculoskeletal Pain on Health-Related Quality of Life Among Adults in Saudi Arabia

**DOI:** 10.7759/cureus.57053

**Published:** 2024-03-27

**Authors:** Majdi Hashem, Reem A AlMohaini, Turki Melfi Alharbi, Muhamad Muslim Aljurfi, Saad Abdullah Alzmamy, Fahad Sulaiman Alhussainan

**Affiliations:** 1 Surgery, Imam Mohammad Ibn Saud Islamic University, Riyadh, SAU; 2 Orthopedic Surgery, King Fahd University Hospital, Dammam, SAU; 3 Internal Medicine, College of Medicine, Imam Mohammed Ibn Saud Islamic University, Riyadh, SAU; 4 Orthopedics, College of Medicine, Imam Mohammed Ibn Saud Islamic University, Riyadh, SAU; 5 Urology, Security Forces Hospital, Riyadh, SAU

**Keywords:** saudi arabia, quality of life, pain, musculoskeletal, disability

## Abstract

Background

Musculoskeletal pain (MSP) is widely recognized as a prevalent public health issue that affects individuals of various genders and age groups. The aim of this study is to assess the influence of musculoskeletal pain on the quality of life (QoL) of adult individuals living in Saudi Arabia.

Method

This is a cross-sectional study using an online-administered questionnaire that was distributed via online platforms in Saudi Arabia for the duration between January and August 2023. Three questionnaire instruments were used in this study. The 36-item Short Form Health Survey questionnaire (SF-36), The 5-level EQ-5D version (EQ-5D-5L), and Roland-Morris Disability Questionnaire. Binary logistic regression analysis was used to identify predictors of better QoL and having mild to moderate disability.

Results

A total of 8359 participants were involved in this study. The most commonly reported sites of musculoskeletal pain were the lower back, neck, and shoulder, accounting for 36.8% (n= 3072), 30.5% (n= 2549), and 30.1% (n= 2514), respectively. The mean pain score for the study participants was 4.3 (SD: 2.3), which indicates mild degree of pain. The median EQ-5D-5L index value for the study participants was 0.827 (0.756-1.00), which demonstrates a high quality of life. The mean SF-36 score for the study participants was 63.11 (17.4), which demonstrates moderate quality of life. The median Roland-Morris Disability score for the study participants was 1.00 (0.00-7.00), which demonstrates a low level of pain-related disability. Male gender, younger age (30-39 years), having higher education attainment, having higher monthly income (more than SAR 20000), and having lower BMI (less than 25.8 kg/cm^2^) were predictors of better QoL (p<0.05).

Conclusion

Musculoskeletal pain is a multifactorial condition influenced by structural, physical, psychological, social, lifestyle, and comorbid health elements. It ranges from acute to chronic pain and, despite rarely being fatal, has a significant impact on QoL. Musculoskeletal discomfort varies in terms of intensity, affected regions, and demographic and lifestyle factors. This study sheds light on the multifaceted nature of MSP, its impact on QoL, and the significance of early intervention and individualized management strategies to improve the QoL of those affected.

## Introduction

Musculoskeletal pain (MSP) refers to the sensation of pain experienced in several anatomical structures, including muscles, ligaments, tendons, nerves, bones, and joints. Musculoskeletal pain is widely recognized as a prevalent public health issue that affects individuals of various genders and age groups. Among the various types of pain, MSP is notably regarded as the most frequently reported. The fundamental factor driving the need for consultation in primary care is well-recognized to be the MSP [1-3]. 

MSP has been found to have a notable impact on individual work activity, resulting in reduced productivity among patients when compared to individuals without medical conditions. In addition to its cost-effectiveness in treatment and management, this factor is also recognized as a prominent contributor to early retirement in some occupations [4]. Furthermore, it is identified as a primary source of work-related health issues in Europe, impacting a wide range of employees across various sectors [5].

Based on the findings of the European Working Conditions Survey conducted in 2015, previous literature indicates that a total of 612 workers were included in the study. The results revealed that three out of five workers experienced MSP in different parts of their bodies. The most commonly reported site of MSP was the back, followed by the upper limbs [5]. According to a study conducted in Sweden, it was found that a significant proportion, namely two-thirds, of pain complaints reported in Swedish primary clinics are attributed to MSP [6]. In the United Kingdom, it is widely acknowledged that sickness absence is a prevalent issue, ranking as the second most common reason [7].

Based on the existing body of literature, it has been observed that the lower back is the most prominently impacted body region, accounting for 25% of reported cases. Following this, the neck exhibits a prevalence of 18%, while both the knees and shoulders demonstrate a comparable incidence of 17%. According to the World Health Organization (WHO) Global Burden of Disease research, neck discomfort and other musculoskeletal illnesses were ranked 4th and 10th, respectively [8, 9]. The prevalence of pain in the neck, lower back, shoulder, elbow, and wrist is higher among individuals aged 45-64, but pain in the hip, knee, ankle, and foot is more prevalent in older age cohorts [8]. Conversely, it has been found that osteoarthritis of the hip, osteoporosis, rheumatoid arthritis, and fibromyalgia exhibit the lowest levels of health-related quality of life (QoL). Nevertheless, individuals diagnosed with MSP have a diminished quality of life in terms of their health compared to the general population [10].

According to a study conducted by Markus Paananen, there exists a correlation between the severity of Health-related Quality of Life (HRQoL) decline in young individuals and the number of affected areas of self-reported musculoskeletal pain [11]. In 2015, a study was undertaken to examine the influence of musculoskeletal pain, among other factors, on the HRQoL of a specific population of workers in the fishing sector, specifically shellfish gatherers in Galicia, Spain. The findings of the study revealed a significant decrease in the participants' QoL, with musculoskeletal pain intensity, hip-knee pain, lower back pain, functional disability resulting from back pain, and the number of affected musculoskeletal sites all having a detrimental effect on the physical health of the workers [12].

The aim of this study is to assess the influence of musculoskeletal pain on the HRQoL of adult individuals living in Saudi Arabia.

## Materials and methods

Study design

This is a cross-sectional study using an online-administered questionnaire that was distributed via online platforms in Saudi Arabia for the duration between January and August 2023.

Study population

All individuals older than 18 years living in Saudi Arabia and willing to participate in the study formed the study population. Participants who are non-communicative and have an intellectual disability were excluded.

Participants recruitment

Participants were recruited using a convenient sampling technique. The study link was distributed through social media platforms (Facebook, WhatsApp, Instagram, and Twitter).

Study instrument

Participants will be required to fill out a 76-question survey, anonymously. Questions are grouped into five groups each containing multiple questions covering the following aspects: Informed consent, demographic data, past medical history, musculoskeletal pain, and quality of life. Three questionnaire instruments were used in this study. The 36-item Short Form Health Survey questionnaire (SF-36), The 5-level EQ-5D version (EQ-5D-5L), and Roland-Morris Disability Questionnaire. The SF-36 questionnaire is widely utilized as an assessment tool for evaluating Health-Related Quality of Life. The SF-36 assesses eight dimensions: physical functioning (PF), role physical (RP), bodily pain (BP), general health (GH), vitality (VT), social functioning (SF), role emotional (RE), and mental health (MH) [13].

The EQ-5D-5L is a widely used tool in the field of health research and evaluation, designed to provide a comprehensive and standardized description and assessment of individuals' health status. The conceptual framework employed in this study utilizes a descriptive system to delineate the various dimensions of health. These categories include mobility, self-care, usual activities, pain/discomfort, and anxiety/depression [14]. The scoring of the EQ-5D-5L is commonly conducted through the utilization of a valuation set or value set, which is produced from surveys conducted on a population-based level.

The purpose of these surveys is to gather data on the preferences of a subset of the population in order to evaluate the comparative significance of various health conditions. The EQ-5D-5L Crosswalk Index Value Calculator is often used as the predominant value set in the EQ-5D-5L. This calculator is accessible in many countries and places. The value set in question entails the allocation of weights to individual health states, which are determined by the preferences of the broader population.

The Roland Morris Disability Questionnaire is an outcome measure that relies on self-reporting [15]. This test offers a means of assessing the degree of disabilities encountered by those afflicted with low back pain. Subsequently, it has emerged as one of the most extensively employed metrics for assessing the effects of low back pain. The questionnaire comprises a total of 24 statements that pertain to an individual's subjective perceptions of their back pain and the resulting disability. The aforementioned categories encompass several aspects of an individual's well-being, namely physical ability/activity (15 items), sleep/rest (three items), psychosocial (two items), household management (two items), eating (one item), and pain frequency (one item).

Sample size

To calculate the sample size, assuming the prevalence of musculoskeletal pain is 30% in Saudi Arabia and using the following formula, the minimum required sample size is 308:

n= (Z^2×p×(1−p)​)/e^2

Where:

n = required sample size; Z = Z-score corresponding to the 95% confidence level (which is equal to 1.96); p = estimated prevalence of musculoskeletal pain in the population; E = margin of error (which is equal to 0.05).

Statistical analysis

Data were analysed via Statistical Package for the Social Science (SPSS) software, version 28 (IBM Corp., Armonk, USA). Categorical variables were presented using frequency and percentage. Continuous variables were presented using mean and (standard deviation (SD)). The Pearson correlation test was used to examine the correlation between the study variables. One-way ANOVA and Student t-test were used to examine the difference in the mean QoL scores across participants from different demographic characteristics. Binary logistic regression analysis was used to identify predictors of better QoL and having mild to moderate disability. The level of significance was assigned as 5%.

## Results

Table 1 below presents the demographic characteristics of the study participants. A total of 8359 participants were involved in this study. More than half of them (68.1%; 5694) were females and aged 18-29 years (55.5%; n=4637). The vast majority of them (94.5%; n= 7895) were Saudis. Around 54.5% (n=4559) of the participants were single. Half of the participants (50.0%; n=4175) reported that they hold a bachelor’s degree. Around one-third (36.4%; n=3039) of the participants were students. Around 53.7% (n=4491) of the participants reported that their monthly income is less than 5000 Saudi Arabian riyals (SAR). Around 28.6% (n=2394) of them reported that they live in the central area. Around 38.7% (n=3231) of the participants reported that they have comorbidities and 20.2% reported that they are smokers. The mean BMI for the study participants was 25.8 kg/cm2 (SD: 6.7). 

**Table 1:** Participants' demographic characteristics. BMI: Body mass index; SD: Standard deviation; SAR: Saudi Arabia riyal

Variable	Frequency	Percentage
Gender		
Female	5694	68.1%
Age category		
18-29 years	4637	55.5%
30-39 years	1182	14.1%
40-49 years	1494	17.9%
50-59 years	741	8.9%
60-60 years	252	3.0%
70-79 years	36	0.4%
80 years and above	17	0.2%
Nationality		
Saudi	7895	94.5%
Marital status		
Single	4559	54.5%
Married	3450	41.3%
Divorced	233	2.8%
Widowed	117	1.4%
Education level		
Primary school or lower	340	4.1%
High school	2495	29.9%
Diploma	759	9.1%
Bachelor’s degree	4175	50.0%
Higher education	590	7.1%
Occupation		
Student	3039	36.4%
Education sector	1299	15.5%
Office work	907	10.9%
Healthcare provider	569	6.8%
Field work	386	4.6%
Freelancer	359	4.3%
Industry and craft labour	137	1.6%
Unemployed	1664	19.9%
Monthly income		
Less than 5000 SAR	4491	53.7%
5000-10000 SAR	1438	17.2%
10000-15000 SAR	1183	14.2%
15000-20000 SAR	704	8.4%
More than 20000 SAR	543	6.5%
Area of residency		
Central area	2394	28.6%
Northern area	1801	21.5%
Southern area	991	11.9%
Eastern area	1975	23.6%
Western area	1198	14.3%
Comorbidities history		
Yes	3231	38.7%
Smoking status		
Smoker	1686	20.2%
Body mass index (BMI) (mean (SD))	25.8 (6.7)	

Participants’ pain profiles

Table 2 below presents the pain profile of the study participants. The majority of the study participants (85.0%; n=7104) reported that their dominant hand was the right hand. The most commonly reported sites of musculoskeletal pain were the lower back, neck, and shoulder, accounting for 36.8% (n=3072), 30.5% (n=2549), and 30.1% (n=2514), respectively. The mean pain score for the study participants was 4.3 (SD: 2.3), which indicates mild degree of pain. Around half of the study participants (49.8%; n=4163) reported that their pain stayed for less than six weeks. When the participants were asked about their health today, the mean score was 6.3 (SD: 3.0), which indicates moderate health status.

**Table 2:** Participants' pain profile SD: Standard deviation

Variable	Frequency	Percentage
Dominant hand		
Right hand	7104	85.0%
Left hand	699	8.4%
Both hands	555	6.6%
Have you ever had pain or discomfort in:		
Lower back	3072	36.8%
Neck	2549	30.5%
Shoulder	2514	30.1%
Knee	2103	25.2%
Upper back	1724	20.6%
Ankle/foot	1529	18.3%
Wrist/ Hand	1207	14.4%
Hip/ Thigh	1162	13.9%
Elbow	520	6.2%
Mean pain score (between 0, representing “no pain”, and 10, representing “the worst pain imaginable”)	4.3 (2.3)	
For how long do you have this pain		
Less than 6 weeks	4163	49.8%
Between 6 weeks and 6 months	1363	16.3%
More than 6 weeks	2834	33.9%
Please indicate on the scale how your health is TODAY (The best health state you can imaging is marked 0 and the worst health state you can imagine is marked 10) (mean score (SD))	6.3 (3.0)	

Predictors of quality of life and mild to moderate disability due to acute, sub-acute, or chronic low back pain

The median EQ-5D-5L index value for the study participants was 0.827 (0.756-1.00), which demonstrates a high quality of life. The mean SF-36 score for the study participants was 63.11 (17.4), which demonstrates moderate quality of life. The mean QoL scores stratified by demographic characteristics are presented in Table 3. There was a statistically significant difference in the mean QoL scores based on all demographic characteristics except nationality and smoking status (for SF-36 score) (p>0.05). 

**Table 3:** Mean quality of life (QoL) score stratified by demographic characteristics

Variable	Mean QoL using SF-36 score (standard deviation)	p-value	Mean QoL using EQ-5D-5L index value (standard deviation)		p-value
Gender					
Female	61.1 (17.0)	<0.001	0.81 (0.17)		<0.001
Males	67.4 (17.3)		0.84 (0.17)		
Age category					
18-29 years	63.5 (17.0)	<0.001	0.82 ()		<0.001
30-39 years	66.2 (17.4)		0.84 ()		
40-49 years	61.5 (18.0)		0.81 ()		
50-59 years	61.1 (16.7)		0.80 ()		
60-60 years	60.0 (17.9)		0.77 ()		
70-79 years	54.8 (18.6)		0.73 ()		
80 years and above	42.6 (17.2)		0.53 ()		
Nationality					
Non-Saudi	63.7 (17.1)	0.455	0.83 (0.16)		0.305
Saudi	63.1 (17.4)		0.82 (0.17)		
Marital status					
Single	63.4 (17.1)	<0.001	0.82 (0.17)		<0.001
Married	63.0 (17.7)		0.82 (0.17)		
Divorced	62.2 (16.7)		0.79 (0.18)		
Widowed	56.3 (19.4)		0.76 (0.17)		
Education level					
Primary school or lower	56.5 (19.3)	<0.001	0.76 (0.22)		<0.001
High school	63.3 (16.6)		0.82 (0.16)		
Diploma	61.6 (17.8)		0.81 (0.17)		
Bachelor’s degree	63.4 (17.2)		0.82 (0.17)		
Higher education	66.2 (18.4)		0.83 (0.17)		
Occupation					
Student	67.5 (18.7)	<0.001	0.84 (0.18)		<0.001
Education sector	60.3 (17.6)		0.81 (0.15)		
Office work	61.9 (18.2)		0.78 (0.22)		
Healthcare provider	63.2 (16.7)		0.82 (0.16)		
Field work	65.6 (17.6)		0.83 (0.17)		
Freelancer	67.0 (17.0)		0.83 (0.17)		
Industry and craft labour	64.7 (17.0)		0.83 (0.17)		
Unemployed	61.2 (17.5)		0.80 (0.18)		
Monthly income					
Less than 5000 SAR	62.5 (17.1)	<0.001	0.81 (0.17)		0.002
5000-10000 SAR	63.1 (18.0)		0.82 (0.17)		
10000-15000 SAR	62.5 (17.7)		0.82 (0.16)		
15000-20000 SAR	68.9 (16.7)		0.82 (0.15)		
More than 20000 SAR	63.1 (17.4)		0.84 (0.16)		
Area of residency					
Central area	66.0 (17.4)	<0.001	0.83 (0.16)		<0.001
Northern area	62.0 (17.7)		0.82 (0.17)		
Southern area	61.4 (16.7)		0.81 (0.17)		
Eastern area	62.9 (17.0)		0.81 (0.17)		
Western area	61.0 (17.3)		0.81 (0.16)		
Comorbidities history					
No	67.3 (16.3)	<0.001	0.85 (0.15)		<0.001
Yes	56.5 (16.9)		0.76 (0.17)		
Smoking status					
Non-smoker	63.0 (17.2)	0.479	0.82 (0.16)		0.008
Smoker	63.4 (18.1)		0.81 (0.18)		
Body mass index categories					
Less than 25.8 kg/cm^2^	64.0 (17.2)	<0.001		0.82 (0.17)	0.007
25.8 kg/cm^2^ and above	62.0 (17.5)			0.81 (0.16)	

Pearson correlation coefficient test showed that there is a moderate positive correlation between EQ-5D-5L index value and SF-36 score for the study participants (r=0.49; 95% confidence interval: 0.48 to 0.51).

The median Roland-Morris Disability score for the study participants was 1.00 (0.00-7.00), which demonstrates a low level of pain-related disability. Pearson correlation coefficient test showed that there is a moderate negative correlation between the SF-36 score and disability score (r=0.51; 95% confidence interval: -0.53 to -0.50). Besides, there is a weak negative correlation between EQ-5D-5L index value and disability score (r=0.38; 95% confidence interval: -0.40 to -0.36).

Binary logistic regression analysis showed that male gender, younger age (30-39 years), having a higher education attainment, having a higher monthly income (more than 20000 SAR), and having a lower BMI (less than 25.8 kg/cm2) were predictors of better QoL (p<0.05). On the other hand, female gender, older age (40 years and above), being married, having a bachelor’s degree, working in the education sector or being unemployed, having a moderate monthly income (SAR 10000-20000), living in areas other than the central area, having comorbidities, and having higher BMI (25.8 kg/cm2 and above) were predictors of higher disability score (p<0.05) (Table 4).

**Table 4:** Binary logistic regression analysis. *p<0.05; **p<0.01, ***p<0.001 QoL: quality of life; SAR: Saudi Arabian Riyal

Variable	Odds ratio of having better QoL using SF-36 score (95% confidence interval)	Odds ratio of having better QoL using EQ-5D-5L index value (95% confidence interval)	Odds ratio of having mild to moderate disability due to acute, sub-acute, or chronic low back pain (95% confidence interval)
Gender			
Female (Reference category)	1.00	1.00	1.00
Male	1.85 (1.68-2.03)***	1.66 (1.51-1.82)***	0.58 (0.53-0.64)***
Age category			
18-29 years (Reference category)	1.00	1.00	1.00
30-39 years	1.34 (1.17-1.52)***	1.28 (1.13-1.46)***	1.04 (0.92-1.18)
40-49 years	0.78 (0.69-0.87)***	0.79 (0.70-0.89)***	1.67 (0.148-1.88)***
50-59 years	0.71 (0.61-0.83)***	0.59 (0.51-0.69)***	1.92 (1.63-2.26)***
60-60 years	0.74 (0.57-0.95)*	0.43 (0.33-0.57)***	1.66 (1.27-2.17)***
70-79 years	0.56 (0.29-1.09)	0.40 (0.20-0.82)*	1.25 (0.64-2.43)
80 years and above	0.12 (0.03-0.51)**	0.12 (0.03-0.53)**	0.79 (0.31-2.06)
Nationality			
Non-Saudi (Reference category)	1.00	1.00	1.00
Saudi	0.99 (0.82-1.20)	1.06 (0.88-1.28)	1.28 (1.06-1.54)*
Marital status			
Single (Reference category)	1.00	1.00	1.00
Married	0.91 (0.83-0.99)*	0.86 (0.79-0.94)**	1.60 (1.46-1.75)***
Divorced	0.93 (0.72-1.21)	0.79 (0.61-1.03)	1.14 (0.88-1.49)
Widowed	0.53 (0.36-0.77)***	0.37 (0.25-0.56)***	1.28 (0.88-1.85)
Education level			
Primary school or lower (Reference category)	1.00	1.00	1.00
High school	1.84 (1.45-2.32)***	1.52 (1.21-1.92)***	0.98 (0.78-1.23)
Diploma	1.49 (1.14-1.93)**	1.34 (1.03-1.74)*	1.29 (1.00-1.67)
Bachelor’s degree	1.93 (1.53-2.42)***	1.55 (1.24-1.94)***	1.29 (1.03-1.61)*
Higher education	2.41 (1.83-3.18)***	1.78 (1.36-2.34)***	1.02 (0.78-1.33)
Occupation			
Field work (Reference category)	1.00	1.00	1.00
Education sector	0.48 (0.38-0.61)***	0.55 (0.43-0.69)***	2.06 (1.64-2.60)***
Industry and craft labour	0.57 (0.38-0.84)**	0.60 (0.40-0.88)**	0.91 (0.61-1.34)
Student	0.69 (0.55-0.86)***	0.77 (0.62-0.95)*	1.16 (0.94-1.43)
Office work	0.85 (0.67-1.09)	0.97 (0.76-1.24)	1.30 (1.02-1.65)
Healthcare provider	1.04 (0.79-1.35)	0.83 (0.64-1.08)	1.36 (1.05-1.76)
Freelancer	0.69 (0.51-0.92)*	0.83 (0.62-1.10)	1.00 (0.75-1.33)
Unemployed	0.55 (0.44-0.69)***	0.62 (0.49-0.77)***	1.47 (1.17-1.83)***
Monthly income			
Less than 5000 SAR (Reference category)	1.00	1.00	1.00
5000-10000 SAR	1.00 (0.89-1.13)	1.02 (0.91-1.15)	1.12 (1.00-1.27)
10000-15000 SAR	1.01 (0.89-1.15)	0.97 (0.85-1.10)	1.52 (1.33-1.74)***
15000-20000 SAR	1.06 (0.91-1.25)	1.06 (0.90-1.24)	1.27 (1.08-1.50)**
More than 20000 SAR	1.84 (1.52-2.21)***	1.39 (1.16-1.67)***	0.96 (0.80-1.15)
Area of residency			
Central area (Reference category)	1.00	1.00	1.00
Northern area	0.63 (0.56-0.72)***	0.89 (0.79-1.01)	1.31 (1.16-1.48)***
Southern area	0.63 (0.54-0.73)***	0.73 (0.63-0.85)***	1.31 (1.13-1.53)***
Eastern area	0.74 (0.65-0.83)***	0.73 (0.65-0.83)***	1.31 (1.16-1.47)***
Western area	0.61 (0.53-0.70)***	0.68 (0.60-0.79)***	1.56 (1.35-1.80)***
Comorbidities history			
No (Reference category)	1.00	1.00	1.00
Yes	0.33 (0.30-0.36)***	0.32 (0.29-0.35)***	2.18 (1.99-2.39)***
Smoking status			
Non-smoker (Reference category)	1.00	1.00	1.00
Smoker	1.06 (0.96-1.18)	1.03 (0.93-1.15)	0.86 (0.77-0.95)**
Body mass index category			
Less than 25.8 kg/cm^2^	1.00	1.00	1.00
25.8 kg/cm^2^ and above	0.82 (0.75-0.89)***	0.80 (0.74-0.88)***	1.43 (1.32-1.56)***

## Discussion

Musculoskeletal pain is a condition characterized by the presence of pain that emerges from a combination of various factors, encompassing structural, physical, psychological, social, lifestyle, and concomitant medical conditions [16]. In the meantime, it is typically seen that MSP tends to resolve spontaneously. However, it is worth noting that MSP can be unexpectedly distressing, exhibiting a spectrum of pain intensity that spans from acute to chronic [17]. Although the fatality rate is typically insignificant in these circumstances, it significantly influences healthcare costs and expenses, as well as disability rates and quality of life [18]. Musculoskeletal pain can vary in severity and location, and is influenced by various factors such as age, gender, education, and lifestyle. MSP can have a profound impact on QoL. Therefore, the aim of this study is to assess the effect of musculoskeletal pain on HRQoL among adults in Saudi Arabia.

The findings of the study indicate that lower back pain is the most frequently reported site of MSP, accounting for 36.8% of cases. This is followed by neck pain at 30.5% and shoulder pain at 30.1%. It is worth noting that low back pain is a significant contributor to disability and limitations in daily activities. Its prevalence is notably high in both developed and developing countries [19]. For instance, the prevalence rates of lower back pain in various Western countries differ, with rates of 14% in the UK, 28.4% in Canada, 33% in Belgium, and 6.8% in North America [20]. However, a review of global data on lower back pain prevalence suggests that developing nations generally exhibit lower rates compared to developed nations. Nevertheless, the review did not establish whether these discrepancies may be attributed to demographic, cultural, or research technique issues [21].

In the context of low back pain, there are multiple elements that have been identified as potential contributors to its incidence. These factors encompass educational level [21], psychological issues [22], as well as occupational factors, namely the physical demands connected with one's employment, which have been found to be associated with a heightened likelihood of experiencing low back pain. According to a previous systematic review, the prevailing occurrence of low back pain among manual employees was found to be 39%, whereas persons with desk-bound employment had a much lower prevalence of 18.3% [23]. A more recent systematic review has revealed several risk factors related to low back discomfort, including manual handling, bending, twisting, and exposure to whole-body vibration [24]. Furthermore, there exists a positive correlation between obesity, defined as having a body mass index (BMI) greater than 30 kg/m2, and an increased probability of encountering low back discomfort [21, 25]. Similarly, there is a notable resemblance between neck and shoulder pain in terms of their incidence risk factors [26] and prevalence, as evidenced by the findings of this study. The occurrence of both types of pain can be attributed to various factors, including work-related activities [27], which have been identified as the primary risk factor for neck pain [28]. The factors that have been found to significantly contribute to the incidence of neck pain are work time, workload, and body posture [29]. In addition, numerous other factors have been found to be correlated with the occurrence of neck and shoulder discomfort. These factors encompass psychosocial elements, such as stress [30], anxiety [31], and depression [29, 32], among others. Additionally, there exists a reciprocal relationship between sleep issues and neck and shoulder pain, whereby the quality of sleep and the occurrence of pain in these areas can mutually influence each other [26, 27].

Neck discomfort is a widely prevalent condition on a global scale, and its occurrence has exhibited a rather consistent pattern from 1990 to 2019, with no notable alterations in its impact [27]. Nevertheless, the findings of this study reveal that the pain score reported by the study participants was 4.3, suggesting a modest level of discomfort. According to the available literature, there is evidence indicating that the intensity and duration of pain have a direct impact on the magnitude of muscle pain [33]. Additionally, muscle discomfort can present in several forms, such as localized in a single place, spread across a certain region, or distributed across the entire body. As the clinical manifestation of pain transitions from one category to another, it is observed to be progressively accompanied by sensory anomalies [17].

The findings of the study indicate that a significant proportion of the participants (49.8%) reported experiencing episodes of pain for a relatively short period, precisely less than six weeks. This finding indicates that a considerable proportion of the individuals experienced episodes of pain that were of a short duration. Chronic pain may occur when its duration surpasses a period of three months [34]. Nevertheless, healthcare practitioners can effectively customize treatment techniques by considering both the duration of pain and the present health status of the individuals involved [35]. For instance, those experiencing acute pain may necessitate distinct interventions compared to those with persistent medical issues. Interventions aimed at alleviating MSP may encompass exercise as a non-pharmacological modality for pain management. Exercise has been shown to ameliorate pain symptoms, reduce physical limitations, and enhance overall well-being [6]. Furthermore, pharmacological strategies, such as corticosteroid injections, nonsteroidal anti-inflammatory drugs (NSAIDs), and opioids, have exhibited efficacy in providing temporary relief for MSP [36]. These techniques have the potential to supplement non-pharmacological treatments.

The research employed a range of instruments to evaluate HRQoL, including the EQ-5D and the SF-36. These instruments facilitate the calculation of utility scores [37, 38]. The researchers also utilized the Roland-Morris Disability Questionnaire, a specific instrument designed to evaluate the level of disability related to low back pain [39].

Our research participants demonstrated an excellent quality of life, as evidenced by the median EQ-5D-5L index value of 0.827. The obtained score indicates that, on average, the participants expressed a reasonably high level of general well-being. On the other hand, the average SF-36 score was recorded as 63.11, suggesting a moderate level of QoL among the individuals involved in the study. In the study, it was observed that the median Roland-Morris Disability score among the participants was 1.00, indicating a lower degree of disability associated with pain. Furthermore, the study revealed a modest to moderate inverse relationship between the QoL score and the disability score. This connection highlights the association between the perceived QoL and pain-related impairment, indicating an inverse link where an increase in one variable is typically accompanied by a reduction in the other. A study conducted in Saudi Arabia examined the health-related quality of life of workers with and without MSP using the SF-36 [40]. The findings indicated that workers with MSP reported significantly lower QoL. These results underscore the significance of managers prioritizing employees who are dealing with MSP, in order to alleviate or prevent its occurrence.

This study revealed a number of demographic and socioeconomic variables that had significant associations with improved QoL and elevated disability ratings among the individuals involved. The aforementioned parameters offer valuable perspectives on prospective domains for focused interventions. It was observed that a higher QoL was linked to male gender, a younger age range (30-39 years), a higher level of education, a higher monthly income (exceeding SAR 20,000), and a lower BMI (below 25.8 kg/cm2). Nevertheless, there exists a positive association between higher disability scores, which signify greater limitations related to pain, and certain demographic factors. These factors include being female, being of older age (40 years and above), being married, possessing a bachelor's degree, working in the education sector or being unemployed, having a moderate monthly income (ranging from SAR 10,000 to 20,000), residing in areas outside the central region, having comorbidities, and having a higher BMI (25.8 kg/cm2 and above).

Multiple socio-demographic characteristics have been found to be connected with MSP. Existing research indicates that there is a clear correlation between female gender and advanced age, and a notable increase in the prevalence of MSP [34]. The higher prevalence of MSP with advancing age can be attributed to physiological changes associated with the aging process, such as postural issues, reduced flexibility, increased musculoskeletal degeneration, and consequent pain exacerbation [41]. However, the greater prevalence and disability of MSP in females can be attributed to factors such as BMI and differences in muscle-to-fat ratio between males and females [42, 43].

In contrast to our research findings, a study conducted in Turkey revealed no significant correlation between educational attainment and the occurrence of low back pain, which was the most frequently reported MSP in our study [44]. Nevertheless, our results align with studies conducted in France [45], Qatar [46], and Austria [47], which indicated that individuals with lower levels of formal education were more likely to engage in strenuous physical activities, had limited access to health-related information, and demonstrated reduced inclination to participate in health-related activities [48-50]. Additionally, it has been observed that there is an association between BMI and the occurrence of MSP. Elevated BMI levels have the potential to modify adipose tissue and metabolic syndrome, leading to significant changes in chronic inflammatory processes that have an impact on the development and progression of several chronic diseases, including MSP [51]. In addition, there exists an inverse correlation between the presence of MSP and the lower-income population. These demographic encounters difficulties in accessing health-related services and exhibits a decreased inclination to participate in health-related activities, consequently limiting their ability to obtain preventive or treatment measures for MSP [48, 49].

This study has limitations. The cross-sectional study design restricted the ability to examine the causality among the study variables. The use of an online survey might have restricted the generalizability of our study findings. Therefore, our findings should be interpreted carefully.

## Conclusions

Musculoskeletal pain is a multifaceted condition that is influenced by various factors, including structural, physical, psychological, social, lifestyle, and concomitant health components. The spectrum of pain ranges from acute to chronic, and while it is typically not life-threatening, it has a substantial influence on QoL. Musculoskeletal pain has a range of severity and is characterized by its variable impact on different anatomical regions. Additionally, this type of pain is subject to the influence of demographic and lifestyle factors. The present study provides insight into the complex characteristics of MSP, its influence on QoL, and the significance of timely intervention and personalized management approaches in improving the welfare of those experiencing this condition.
